# “We want to hug a friend”: the covid-19 pandemic among low-income adolescents

**DOI:** 10.11606/s1518-8787.2023057004778

**Published:** 2023-05-11

**Authors:** Cristiane da Silva Cabral, Jamile Silva Guimarães, Amanda Teixeira, Nicolas Kimura Generoso, Ivan França, Ana Luiza Vilela Borges

**Affiliations:** I Universidade de São Paulo Faculdade de Saúde Pública Departamento Saúde, Ciclos de Vida e Sociedade São Paulo SP Brasil Universidade de São Paulo. Faculdade de Saúde Pública. Departamento Saúde, Ciclos de Vida e Sociedade. São Paulo, SP, Brasil; II Universidade de São Paulo Faculdade de Medicina São Paulo SP Brasil Universidade de São Paulo. Faculdade de Medicina. São Paulo, SP, Brasil; III Universidade de São Paulo Faculdade de Saúde Pública Programa de Pós-Graduação em Saúde Pública São Paulo SP Brasil Universidade de São Paulo. Faculdade de Saúde Pública. Programa de Pós-Graduação em Saúde Pública. São Paulo, SP, Brasil; IV Universidade de São Paulo Faculdade de Saúde Pública São Paulo SP Brasil Universidade de São Paulo. Faculdade de Saúde Pública. Graduação em Nutrição. São Paulo, SP, Brasil; V Universidade de São Paulo Escola de Enfermagem Departamento de Enfermagem em Saúde Coletiva São Paulo SP Brasil Universidade de São Paulo. Escola de Enfermagem. Departamento de Enfermagem em Saúde Coletiva. São Paulo, SP, Brasil

**Keywords:** Adolescent, Emotional Adjustment, Interpersonal Relations, Social Behavior, Education, Distance, COVID-19

## Abstract

**OBJECTIVE:**

To examine the perceptions of adolescent students from a public school, of both sexes, living in a peripheral region of the city of São Paulo, Brazil, in relation to the covid-19 pandemic, with a special focus on their experiences regarding education and sociability.

**METHODS:**

This study is part of the Global Early Adolescent Study. Seven face-to-face focus groups were conducted with adolescents between 13 and 16 years old (19 girls and 15 boys) in 2021.

**RESULTS:**

The experience of remote teaching was frustrating for the adolescents, without the daily and personalized monitoring of the teacher(s). In addition to the difficult or impossible access to devices and the lack of support from schools, there is also the domestic environment, which made the schooling process more difficult, especially for girls, who were forced to take on more household and family care tasks. The closed school blocked an important space for socialization and forced family interaction, generating conflicts and stress in the home environment. The abrupt rupture brought feelings of fear, uncertainty, anguish and loneliness. The iterative evocation of the words “stuck”, “alone” and “loneliness” and the phrase “there was no one to talk to” shows how most of the adolescents experienced the period of distancing. The pandemic aggravated the objective and subjective conditions of preexisting feelings, such as “not knowing the future” and the prospects of a life project.

**CONCLUSION:**

It has been documented how pandemic control measures implemented in a fragmented way and without support for the most impoverished families have negative effects on other spheres of life, in particular for poor young people. The school is a privileged territory to propose/construct actions that help adolescents to deal with problems aggravated in/by the pandemic.

## INTRODUCTION

Two years after the first case of covid-19 infection in Brazil, many gaps still remain to be filled in scientific knowledge, especially with regard to the various ruptures experienced by different generations, genders and social segments. The covid-19 pandemic was especially devastating for impoverished urban communities living in high-density areas, with limited access to such resources as public services, region/neighborhood infrastructure, and disposable income to sustain lockdowns (in particular, individual and collective non-pharmacological measures) related to contagion and illness^1^.

The closing of schools was one of the first non-pharmacological measures adopted in the face of the covid-19 pandemic – and the longest lasting one –, having a major negative effect on the learning process. The return to face-to-face activities in schools was marked by several protocols with different intervention measures. Our understanding of how adolescents from different contexts (social, local and global) have experienced and are still facing ruptures in educational, family and social terms requires in-depth and transversal analysis.

Unlike adults, who are more subject to severe forms of the disease, adolescents seem to be more affected in terms of psychosocial health and emotional well-being^2-4^, mainly due to the deleterious effects of the suspension of face-to-face classes and closure of schools on the health and well-being of children, adolescents and young people^5-7^.

The social and economic crisis generated by the pandemic exacerbates social inequalities, which tend to worsen/degrade the living conditions of those living in poor communities, altering their educational aspirations, health and well-being. Understanding the individual, relational, family and social effects, in the short, medium and long term, associated with the pandemic context, on the trajectories of adolescents from poor urban communities is one of the objectives of the international multicenter Global Early Adolescent Study (Geas), conducted in eleven countries from different continents, including Brazil^8,9^.

This article is part of this initiative, by analyzing the results of the first phase of the research “Covid-19 pandemic and adolescents: challenges faced from physical and social distancing measures”. Here, the perceptions of adolescents of both sexes living in a peripheral region of the city of São Paulo (SP - Brazil) are examined in relation to the covid-19 pandemic, with a special focus on their experiences regarding education and sociability. It is argued here that the school is also an important space for diverse learning and sociability, as well as a privileged territory for interventions of a structural and collective nature to face social and health inequalities, deepened by the pandemic.

## METHODS

This article presents the results produced in the first round of the qualitative stage of the Geas-covid study in Brazil. Focus groups (FG) were conducted in September 2021, on the premises of a public school located in an impoverished area in the far east of the city of São Paulo – a region where the Geas study has been developing since 2018. That is a location which, until the early 1980s, corresponded to a more ruralized area with low real estate value. The fast and disorganized growth is observed in the cluster of slums in the territorial expansion of the neighborhood; there are popular housing complexes (new or old), large two-floor houses in more wooded areas, villas with houses without external cladding and even wooden shacks.

Seven mixed FGs were carried out, with students between 13 and 16 years old. In total, we had 34 participants; of these, 19 were girls and 15 were boys – an average of 5 adolescents in each FG. The FGs were held in a meeting room provided by the school’s coordination. A quiet space, with a large and long table, accompanied by padded chairs, ensured both comfort and privacy and secrecy of conversations. The 7 FG were conducted by the same moderator, accompanied by an observer, who was responsible for recording comments and non-verbal communications. All sessions were recorded, fully transcribed and lasted an average of 50 minutes. Each FG had participants from different classes, but all were enrolled in the same grade; we also sought to establish gender parity in each session. Students from the eighth and ninth years of elementary school II and the first year of high school participated, in order to meet the age criteria established by the general coordination for this component of the study.

The engagement of the adolescents took place through an invitation and presentation of the research in the classrooms and through mobilization among peers. Research information (objectives, methodological instrument and debate topics) was disseminated to parents and students via the school’s institutional WhatsApp. Contacts for the guardians’ consent were mediated by the school coordination. The presentation – and invitation – of the research was made by the moderator and the observer in the classrooms, the day before and again on the day of each FG. Adolescents clarified group and individual doubts about the research, and confirmed their participation before the start of each FG in writing.

Although initially planned to take place in an online format, the FGs were conducted face-to-face. The impossibility of counting on the adherence of adolescents in the empirical stage of the research in remote mode signaled the structural and logistical difficulties for digital communication with residents of impoverished areas of the city, one of the dimensions that greatly affected the adolescent context.

The first round of discussion focused on issues related to adolescents’ experiences of physical and social distancing. The FG script privileged themes such as routine changes, prevention practices adopted against the covid-19 virus, school context, sociability and maintenance of friendship relationships, intra-family dynamics instituted and/or stressed by the context of the pandemic and the impact of covid-19 on the adolescents’ health and well-being. This roadmap was prepared having as its central axis the elements common to the other countries participating in the Geas study.

Thematic analysis of the results was done in order to understand the adolescents’ perceptions and experiences of the pandemic. The theoretical perspective proposed by Fiorin^10^was adopted, which conceives the narrative as an expression of a network of relationships in which the individual is included. This type of analysis allows understanding the individuals’ world view – understood as the content of their action – and the context in which it is produced. Through and in the discourse, one can apprehend the social, cultural and symbolic dimensions that are expressed in it and that explain the way in which the members of the community that reproduce it relate, behave and act.

The project was approved by the Research Ethics Committee of the Faculty of Public Health of the University of São Paulo (number CAAE 31664720.8.0000.5421). The ethical issues established in Resolution 510/2016, of the National Health Council, were considered throughout the conduct of the study: information about the investigation was previously provided to the participants, and the confidentiality of the conversations and anonymity of all with the use of fictitious names was ensured. Phrases or expressions between quotation marks are fragments of youth narratives, placed here as a way of explaining or exemplifying some dimension under analysis. All names used in this article are fictitious.

## RESULTS AND DISCUSSION

### “They passed me out of pity”: challenges of the teaching-learning process from emergency remote teaching

One of the first safety and prevention measures for infections by the covid-19 virus was the establishment of physical and social distancing, which implied restriction and/or interruption of face-to-face activities in various spaces. On March 17, 2020, the Ministry of Education authorized the holding of remote/online classes in all teaching units, from basic to higher education^11^.

Emergency remote teaching (ERE) is an approximation of distance learning (EAD). In ERE, schools could resort to any technological means (blogs, websites, electronic mail, virtual learning environment – AVA) that made communication viable and ensured the continuity of teaching. Until then, the distance learning mode and the information and communication technologies (ICT) occupied a marginal space and role in the national educational system. There was a shortage of investment in technological and continuing education apparatus for teachers to use virtual environments for teaching^12^.

In 2020, the Department of Education of the State of São Paulo (SEDUC-SP) launched the São Paulo Education Media Center (CMSP), which objective was to ensure the maintenance of classes, without major damage to the school calendar, in the period of social confinement. The CMSP brings together a virtual learning space available in the form of an application for mobile devices such as cell phones and tablets, as well as a television transmission system on open channels for the state of São Paulo. Students would be able to access their respective classes and activities and interact live with each other and with teachers through the app. Those who did not have a mobile device could watch the classes of all subjects directly on television at specific times. Thus, both accesses have synchronous and asynchronous classes taught by public school teachers.

The research participants considered the experience of remote teaching, including the Media Center, to be quite frustrating and precarious: there were constant and emphatic criticisms of the disconnection between the contents and the lack of interaction between teachers and students in this application. Mostly, they considered studying as the biggest challenge of the pandemic period (even more than surviving). Also, the transfer of the teaching-learning context to the domestic scope weighed more than the disconnection between teacher and student.

Remote teaching exposed and intensified social inequalities associated with exclusion or digital literacy that affect economically disadvantaged segments, such as the lack of technological devices like computers, tablets or smartphones with adequate processors and memory and precarious (or non-existent) access to the internet^13^. Some students reported not being able to study because they did not have access to the internet and/or a cell phone – “I couldn’t study, they passed me out of pity” (Vanessa, 15 years old). These barriers to good study conditions were not sufficiently mitigated by the education network, without the necessary investment to purchase computers, laptops and the installation of compatible broadband. Even though the school remained open for some periods, adolescents who attended online classes reported difficulties in attending them because there was not enough equipment (computers and laptops) or an adequate internet network (Wi-Fi) to meet the demands in the first year of the pandemic (year 2020).

The centralization of online education in app’s made student duties more flexible: it blurred, for example, obligations with meeting deadlines for delivering work, fundamental for the constitution of a discipline in study and the effectiveness of the practice of studying. Equally problematic were the generic contents, distanced from the students’ knowledge and objectively devoid of context, offered in classes promoted by the state department of education. Standardized teaching brought them added difficulty, as it was not compatible with their learning level. The students also pointed out differences in language and content in relation to the usual communication and themes presented by their teachers as barriers to learning. According to the reports, there is a clear confusion regarding the activities and the various digital communication tools and platforms that have been used throughout the pandemic “confinement”.

Another aspect was the impossibility for the teachers to follow the development of the students, since they did not have access to the activities carried out in the Media Center. The conduct of distance learning by the government was considered “very bad”, since there was no gradual development of aspects, elements and contexts related to the themes under study, as done by the school teachers. In online classes:

“they go straight to the point… they just want the result… ask here and want me to answer there. They are doing as if we had studied it before. There was no recap in the next class like there is at school. Interlocution in the classroom allows the teacher to realize that the student did not understand, one goes back to the previous content, until they understand” (Téo, 15 years old).

Being at home full time seems to have amplified the adolescents’ household tasks, making the schooling process more difficult, especially for girls^14,15^. Having to “take care of the brother, take care of the house” brought difficulties in concentrating on studies, with delays in schoolwork and even more “discouragement” with the context experienced. This enlargement of responsibilities for taking care of younger siblings and cleaning the house for girls signals the permanence of the traditional sexual division of domestic work. In general, boys had fewer tasks, such as “washing dishes and sweeping”; they took up cleaning only when they were home alone or when they were the eldest child with younger siblings. This fact is highlighted by the way some boys referred to the tasks, describing them as a “favor” asked by the mother, not a consolidated obligation.

### “We Want to Hug a Friend”: Breaks in Youth Sociability

The suspension of face-to-face teaching brought the teenagers an interruption of social relationships and important bonds experienced in the school environment: the school is a territory of protection, food and sociability^16^. Before the pandemic, Sara used to get together with her friends to go to the neighborhood square “to have an ice cream”, “and today you can’t anymore, there’s this distance. If you are like this [close] they say ‘oh, the distance!’, ‘you are very close to each other!’. So this sucks. We want to hug a friend. We don’t like hugging our family at home…” (Sara, 16 years old).

Social networks played a key role in maintaining socialization between peers^17,18^, but face-to-face contact creates a network of affection, hugs and conversations that online contact does not allow. Friends are a significant support among young people, in a constitutive process of this phase of life in which the separation from the family nucleus is intensified and the peer group gets closer together^19^. Coping with the separation was “very hard”, as it happened precisely at a time in the course of life when adolescents are eager to find autonomy and identity^19,20^. Although they could use social networks – Instagram, WhatsApp, Facebook – to communicate virtually with friends, the “preference to talk in person” was the keynote of the discourses, justified by the need for “physical contact” or by the “little patience” for the virtual communication, as expressed in the statement “I have no patience for WhatsApp” (Renata, 15 years old).

Young people began to spend more time with family members, who also had their routine interrupted due to enforced health rules, especially in the first year of the pandemic. Many fathers and mothers had their jobs changed, either because they started to work remotely, or because they became unemployed. Spending more time with family members was identified as a generator of conflicts, stress and violence in the family environment, which has implications for the well-being and health of adolescents^21,22^.

The period of adolescence is marked by many changes and also experimentations. The context of the covid-19 health crisis generated barriers to socialization and self-building for adolescents^19^. This rupture, occurring abruptly, brought feelings of fear, uncertainty, anguish, loneliness – very frequent terms in their narratives. “The pandemic pushed people away, it generated loneliness and emptiness because I was trapped, so there was a strange, dark, heavy atmosphere. And we end up feeling bad” (Luiza, 16 years old). In another FG, some of the participants report the centrality of feelings of uncertainty and fear:

Moderator: How do you think the pandemic contributed to this anxiety crisis?

Yara (15): Not seeing if this will end, not knowing if one day it will end, if one day we will stop wearing a mask.

Simone (16): Not knowing the future.

Yara (15): Now that I’ve had the vaccine, I’m feeling calmer, but before that I was like: “My God, is there going to be a vaccine? Will the world end? Is everyone going to die?”

Renata (15): Yeah, “will I get covid? I wonder if… I wonder what…?”

Yara (15): “Will I die if I get covid?”

Renata (15): No, it wasn’t even me getting [the] covid; like, my mother... My mother has asthma, she has... she has everything, right? And then, “what if I pass it on to my mother? will she die?” Because if I get it, okay, but what about my mother? My father? My brother? My grandma?

The iterative evocation of the words “stuck”, “alone” and “loneliness” and the phrase “there was no one to talk to” sets the tone for how most adolescents experienced the period of physical and social distancing. The condition of being alone is an unintentional result of long-term containment measures, making the group vulnerable and increasing the risk of developing mental health problems^23^. Even when family relationships are not marked by conflicts, these do not usually make up for the absence of friends, as friendship involves common affinity, interests and styles.

Authors argue about the need for special attention to the shocks resulting from physical-social distancing and the suspension of face-to-face activities for adolescents^24,25^. The old school routine or with activities outside the home was replaced by one in which “boredom” and “discouragement” prevailed. Recurrent reports among adolescents include: “I only slept and ate”, “watched TV”, “played on my cell phone”, “played games”, in addition to increasing household chores. Perceived changes also relate to lower productivity, increased sedentary lifestyle, adoption of eating habits that are less favorable to health and a disrupted sleep routine^23,26^. Research carried out with students in the ninth year of the fundamental level, enrolled in teaching units in the Metropolitan Region of São Paulo, recorded the time of exposure to screens, sleep inversion and being female as elements associated with symptoms of depression and anxiety among study participants^26^.

At this point, it is important to locate the centrality of the school as a space for sociability and the making of affective and emotional bonds between friends. Issues of psychosocial suffering^7^related to family problems (conflicting/violent, negligent relationships and emotional distancing) are present in the narratives. Leaving school means leaving friends, contact, presence, support from schoolmates. Classmates constitute a network of solidarity that provides continuous emotional and affective support. The “group” is able to perceive days of sadness and hopelessness and tries to make the partner happy with conversations and games. This bond strengthens as they get to know each other by advancing through the school years together. Moments of mutual help operate as a space for promoting care and freedom of positioning; they have educational relevance by stimulating social skills and meaning-making about their beliefs, aspirations, fears, anxieties – feelings closely related to the construction of identity^27^.

Keeping adversities to oneself can exacerbate disillusionment, suicidal ideation, depression symptoms and the very feeling of loneliness – dimensions present in the speeches of FG participants and in other moments of interaction, including pre-pandemic, made between study researchers and students from the same school^2^. The frequent narratives about psychosocial distress seem to reiterate the argument of Najar and Castro^29^who, recovering another author, Frank Furedi, talk about the psychologization of everyday life and a greater sense of existential insecurity as some of the elements of a certain “cultural script” that would be influencing the reactions to covid-19. For these authors, “the way a social group responds to a threat, such as a pandemic, is mediated by the perception of the threat, its sense of existential security and the ability to make sense of unpredictable experiences” (p. 145).

### The “World’s Greatest Psychological Experiment”: Feelings in the Face of the Extraordinary

The state of social contingency introduced by the pandemic aggravated the pre-existing conditions of feelings of anxiety. The uncertainties, the “not knowing the future”, break with perspectives of life project. “Fear”, added to excessive “worry”, and being “alone” are constants experienced and reported by adolescents. The girls offered more detailed reports about these feelings, while the boys restricted themselves to expressing “anger” and “hatred” for the disruption of their school routine and interaction with friends. Similar reports and situations are found in other studies with adolescents and young people: being “confined” imposed by the pandemic was described by the participants of this study, as well as in the study by Lima^30^, as the “greatest psychological experiment in the world”,^,^placing in question the human capacity to find some meaning in the midst of so much suffering and challenges, individual and collective.

Confined to a home space and with no leisure activities, some of the adolescents reported that they slept to pass the time and avoid boredom. They also stated that they became “more nervous” during the pandemic and that they gained weight – “I gained about 10 kg in the quarantine” (Vanessa, 15 years old); “I gained 11 kg” (Mônica, 15 years old). The lack of entertainment options at home, combined with the decrease in physical activity, increased sedentary lifestyle, “stress” and “anxiety”, helps to understand the weight gain reported by girls^24^.

Some boys have similar reports to those of girls, such as sleeping or staying in their room playing all day. However, there are those who repeatedly break the period of physical distancing to be with close friends and classmates (neighbors) or girlfriends/lovers, emphasizing that they did so when there was a reduction in the number of cases of infection and deaths. It should be noted that non-pharmacological measures were often adhered to by young people due to their mothers’ demand and vigilance. However, those whose mothers remained in face-to-face work did not strictly comply with the period of distancing: the eagerness for socializing meant that there was an escape from isolation.

Also, the youth narrative associates constant fear and insecurity about the disease and death of family members with feelings of depression and anxiety. This type of association is pointed out by the specific literature on mental health: studies indicate that adolescents who had parents in jobs considered essential, or who were unable to maintain physical distance, had more symptoms of psychological distress. Similarly, adolescents who lived in families with vulnerable economic conditions had higher rates of stress^23,31^.

The convergence of the deleterious effects resulting from the ongoing pandemic has negative repercussions in different spheres of life, with emphasis on aspects associated with psychosocial suffering^7,30^. Physical distancing measures brought added concerns to adolescents, whose stage of life is marked by the centrality of the group and potentialization of the gregarious feeling among peers^14^. This has been considered an important trigger for the emergence of problems related to psychosocial suffering, enhanced by situations of individual, social and programmatic vulnerability of adolescents in the face of the chaotic pandemic scenario^7^.

## FINAL REMARKS

Measures of physical and social distancing and the shutting down of schools harmed the process of schooling and socialization of young people, as well as limited important opportunities for peer interaction, with an impact on the adolescents’ development of identity, autonomy and independence and on maintaining physical and psychosocial health. This study documents how measures to control the pandemic, implemented in a fragmented way and without support for the most impoverished families, have negative effects on other spheres of life, in particular for poor young people.

This research shows the implications and weaknesses of adolescents living in a peripheral and very vulnerable region in a large Latin American metropolis. The social context proved to be decisive for this group’s experience of the covid-19 pandemic, from intermittent adherence to non-pharmacological prevention measures to the very impossibility of carrying out social distancing, since many had family members who were working in services considered essential, with the need to use crowded public transport in the most acute phase of the pandemic.

Adolescents reported feelings of depression and anxiety, generated by the anguish and fear caused by the covid-19 pandemic. Many spoke about their relationship with their friends and the support network they represent. Direct health effects, such as physical inactivity and weight gain, were mentioned. However, it is still too early to fully understand the effects of the pandemic and the long-term implications of the disease control and prevention strategies adopted in Brazil, a country that is among those with the most cases and deaths from covid-19 in the world^32^.

The UN points out that countries must ensure safe access to high-quality education in schools during emergencies with the same attention given to health services^33^. The school is shown as a territory of sociability, support and refuge to deal with family and personal problems, as well as a scenario for identifying abuse and negligence. Apparently, it is also a privileged place to propose/build actions that help these teenagers to deal with the wounds left by the pandemic. “In peripheral areas, both vulnerabilities and opportunities have, to a large extent, territorial bases”^26^. Even though we believe in the resilience and plasticity of children and young people, this research indicates that the road to recover youth teaching and sociability will be long and deserves increased attention^34^. This scenario demands efforts, especially in terms of public policy propositions to face the various social inequalities (including those of gender, social class, race/ethnicity, regions of residence) exacerbated by the pandemic, as well as in relation to the possibilities of recovery^33^, considering the local dynamics of the pandemic.
