# Supplementary material for: “We want to hug a friend”: the covid-19 pandemic among low-income adolescents
Source: Rev Saude Publica. 2023 May 11;57(Suppl 1):5s. doi: 10.11606/s1518-8787.2023057004778 (PMC10185316; doi:10.11606/s1518-8787.2023057004778)
Supplement: Translation

# Translation: “A gente quer abraçar o amigo”: a pandemia de covid-19 entre adolescentes de baixa renda

**Authors:** Cristiane da Silva Cabral, Jamile Silva Guimarães, Amanda Teixeira, Nicolas Kimura Generoso, Ivan França, Ana Luiza Vilela Borges

**Affiliations:** I Brasil Universidade de São Paulo. Faculdade de Saúde Pública. Departamento Saúde, Ciclos de Vida e Sociedade. São Paulo, SP, Brasil; II Brasil Universidade de São Paulo. Faculdade de Medicina. São Paulo, SP, Brasil; III Brasil Universidade de São Paulo. Faculdade de Saúde Pública. Programa de Pós-Graduação em Saúde Pública. São Paulo, SP, Brasil; IV Brasil Universidade de São Paulo. Faculdade de Saúde Pública. Graduação em Nutrição. São Paulo, SP, Brasil; V Brasil Universidade de São Paulo. Escola de Enfermagem. Departamento de Enfermagem em Saúde Coletiva. São Paulo, SP, Brasil

**Keywords:** Adolescente, Ajustamento Emocional, Relações Interpessoais, Comportamento Social, Educação à Distância, COVID-19

## Abstract

**OBJETIVO:**

Examinar as percepções de adolescentes estudantes de uma escola pública, de ambos os sexos, moradores de uma região periférica da cidade de São Paulo, Brasil, em relação à pandemia de covid-19, com especial enfoque em suas experiências quanto à educação e sociabilidade.

**MÉTODOS:**

Este estudo faz parte do *Global Early Adolescent Study*. Foram conduzidos sete grupos focais presenciais com adolescentes entre 13 e 16 anos (19 meninas e 15 meninos),em 2021.

**RESULTADOS:**

A experiência do ensino remoto foi frustrante para as/os adolescentes, sem o acompanhamento cotidiano e personalizado de professoras(es). Ao acesso difícil ou impossível aos dispositivos e à ausência de suporte das escolas é acrescido o ambiente doméstico, que dificultou o processo de escolarização, em especial para as meninas, obrigadas a assumir mais tarefas de cuidado da casa e da família. A escola fechada bloqueou espaço importante de socialização e forçou o convívio familiar, gerando conflitos e estresse. A abrupta ruptura trouxe sentimentos de medo, incerteza, angústia e solidão. A evocação iterativa das palavras “presa/o”, “sozinha/o” e “solidão” e da frase “não teve ninguém pra conversar” mostra como grande parte das/os adolescentes experienciou o período de distanciamento. A pandemia agravou as condições objetivas e subjetivas de sentimentos preexistentes, como “não saber o futuro” e as perspectivas de projeto de vida.

**CONCLUSÃO:**

Foi documentado como medidas de controle da pandemia implementadas de forma fragmentada e sem suporte às famílias mais empobrecidas têm efeitos negativos em outras esferas da vida, em particular para jovens pobres. A escola é um território privilegiado para se propor/construir ações que auxiliem as/os adolescentes a lidar com problemas agravados na/pela pandemia.

## INTRODUÇÃO

Passados dois anos desde o primeiro caso de infecção por covid-19 no Brasil, ainda há muitas lacunas a serem preenchidas pelo conhecimento científico, sobretudo no que concerne às diversas rupturas experimentadas por diferentes gerações, gêneros e segmentos sociais. A pandemia de covid-19 foi especialmente devastadora para as comunidades urbanas empobrecidas habitantes de áreas de alta densidade populacional, com acesso limitado a recursos como serviços públicos, infraestrutura da região/bairro e renda disponível para sustentar bloqueios (em especial, medidas individuais e coletivas de caráter não farmacológico) relacionados ao contágio e ao adoecimento^1^.

O fechamento das escolas foi uma das primeiras medidas não farmacológicas adotadas no enfrentamento da pandemia de covid-19 – e a mais duradoura –, tendo como efeito importante prejuízo no processo de aprendizado. O retorno às atividades presenciais nas escolas tem sido marcado por diversos protocolos com distintas medidas de intervenção. Nosso entendimento sobre como as/os adolescentes de diferentes contextos (sociais, locais e globais) experimentaram e ainda têm enfrentado as rupturas em termos educacionais, familiares e sociais necessita de análises aprofundadas e transversalizadas.

Diferentemente dos adultos, mais sujeitos a formas graves da doença, as/os adolescentes parecem afetar-se mais em termos de saúde psicossocial e do bem-estar emocional^2-4^, principalmente por conta dos efeitos deletérios da suspensão das aulas presenciais e do fechamento das escolas sobre a saúde e bem-estar de crianças, adolescentes e jovens^5-7^.

A crise social e econômica gerada pela pandemia recrudesce desigualdades sociais, que tendem a piorar/degradar as condições de vida daqueles que vivem em comunidades pobres, alterando suas aspirações educacionais, sua saúde e seu bem-estar. Compreender os efeitos individuais, relacionais, familiares e sociais, em curto, médio e longo prazo, associados ao contexto pandêmico, nas trajetórias de adolescentes de comunidades urbanas pobres é um dos objetivos do estudo multicêntrico internacional *Global Early Adolescent Study* (Geas), conduzido em onze países de vários continentes, incluindo o Brasil^8,9^.

Este artigo surge como parte dessa iniciativa, ao analisar os resultados da primeira fase da pesquisa “Pandemia covid-19 e os/as adolescentes: desafios enfrentados a partir das medidas de distanciamento físico-social”. Aqui, são examinadas as percepções de adolescentes, de ambos os sexos, moradores de uma região periférica da cidade de São Paulo (SP – Brasil), em relação à pandemia de covid-19, com especial enfoque em suas experiências quanto à educação e sociabilidade. Argumenta-se aqui que a escola é, igualmente, espaço importante de aprendizagens diversas e de sociabilidade, bem como território privilegiado para intervenções de caráter estrutural e coletivo de enfrentamento às desigualdades sociais e em saúde, aprofundadas pela pandemia.

## MÉTODOS

Este artigo apresenta os resultados produzidos na primeira rodada da etapa qualitativa do estudo Geas-covid no Brasil. Foram conduzidos grupos focais (GF) em setembro de 2021, nas dependências de uma escola pública localizada numa área empobrecida do extremo leste da cidade de São Paulo – região em que o estudo Geas vem se desenvolvendo desde 2018. Trata-se de uma localidade que, até o princípio da década de 1980, correspondia a uma área mais ruralizada e com baixo valor imobiliário. O crescimento rápido e desordenado é observado no aglomerado de favelas na expansão territorial do bairro; há conjuntos habitacionais populares (novos ou antigos), grandes sobrados em áreas mais arborizadas, vilas com casas sem revestimento externo e até barracos de madeira.

Foram realizados sete GF mistos, com estudantes entre 13 e 16 anos. No total, tivemos 34 participantes; destes, 19 eram meninas e 15, meninos – uma média de 5 adolescentes em cada GF. Os GF foram realizados em uma sala de reuniões cedida pela coordenação da escola. Espaço silencioso, com uma mesa grande e comprida, acompanhada de cadeiras acolchoadas, garantiu tanto o conforto, quanto a privacidade e o sigilo das conversas. Os 7 GF foram conduzidos por uma mesma moderadora, acompanhada de uma observadora, que foi responsável pelo registro de comentários e comunicações não verbais. Todas as sessões foram gravadas, integralmente transcritas e tiveram duração média de 50 minutos. Cada GF contou com participantes de diferentes turmas, porém todos/as eram matriculados na mesma série; procurou-se ainda estabelecer uma paridade de gênero em cada sessão. Participaram alunas/os do oitavo e nono anos do ensino fundamental II e do primeiro ano do ensino médio, de forma a atender o critério etário estabelecido pela coordenação geral para esse componente do estudo.

O engajamento dos/as adolescentes deu-se mediante convite e apresentação da pesquisa nas salas de aula e por meio da mobilização entre pares. Procedeu-se à divulgação de informações da pesquisa (objetivos, instrumento metodológico e temas de debate) junto a pais e estudantes, via WhatsApp institucional da escola. Os contatos para o consentimento de responsáveis foram mediados pela coordenação da escola. Foi feita a apresentação – e convite – da pesquisa pela moderadora e pela observadora nas salas de aula, um dia antes e novamente no dia de realização de cada GF. As/os adolescentes tiraram dúvidas em grupo e individualmente sobre a pesquisa, e confirmaram sua participação antes do início de cada GF por escrito.

Embora inicialmente previstos para ocorrer no formato *online*, os GF foram conduzidos de forma presencial. A impossibilidade de contar com a adesão das/os adolescentes na etapa empírica da pesquisa em modalidade remota sinalizou as dificuldades estruturais e de logística para comunicação digital com moradores de áreas empobrecidas da cidade, uma das dimensões que afetou sobremaneira o contexto adolescente.

A primeira rodada de discussão concentrou-se em questões relacionadas às experiências do distanciamento físico-social das/os adolescentes. O roteiro dos GF privilegiou temas como mudanças de rotina, práticas de prevenção adotadas contra o vírus da covid-19, contexto escolar, sociabilidade e manutenção de relacionamentos de amizade, dinâmicas intrafamiliares instituídas e/ou tensionadas pelo contexto da pandemia e impacto da covid-19 na saúde e no bem-estar das/os adolescentes. Tal roteiro foi elaborado tendo como eixo central os elementos comuns aos demais países participantes do estudo Geas.

Com o fito de compreender as percepções e experiências da pandemia vividas pelas/os adolescentes, optou-se pelo procedimento de análise temática dos resultados. Adotou-se a perspectiva teórica proposta por Fiorin^10^, que concebe a narrativa como expressão de uma rede de relações na qual o indivíduo está inserido. Este tipo de análise permite a compreensão da visão de mundo dos indivíduos – entendida como o conteúdo de sua ação – e do contexto em que é produzida. Por meio do e no discurso, pode-se apreender as dimensões sociais, culturais e simbólicas que nele se expressam e que explicam a forma pela qual os membros da comunidade que o reproduzem se relacionam, comportam-se e agem.

O projeto foi aprovado pelo Comitê de Ética em Pesquisa da Faculdade de Saúde Pública da Universidade de São Paulo (número CAAE 31664720.8.0000.5421). As questões éticas estabelecidas na Resolução 510/2016, do Conselho Nacional de Saúde, foram consideradas durante toda a condução do estudo: forneceu-se previamente informações acerca da investigação aos participantes e assegurou-se o sigilo das conversas e anonimato de todos/as com o uso de nomes fictícios. Frases ou expressões entre aspas são fragmentos das narrativas juvenis, colocados aqui como forma de explicitar ou exemplificar alguma dimensão em análise. Todos os nomes utilizados neste artigo são fictícios.

## RESULTADOS E DISCUSSÃO

### “Me passaram por dó”: desafios do processo de ensino-aprendizagem a partir do ensino remoto emergencial

Uma das primeiras medidas de segurança e de prevenção das infecções pelo vírus da covid-19 foi o estabelecimento do distanciamento físico-social, que implicou restrição e/ou interrupção de atividades presenciais em diversos espaços. Em 17 de março de 2020, o Ministério da Educação autorizou a execução de aulas remotas/online em todas as unidades de ensino, desde a educação básica ao ensino superior^11^.

O ensino remoto emergencial (ERE) é uma aproximação do ensino à distância (EAD). No ERE, as escolas poderiam recorrer a qualquer meio tecnológico (blogs, sites, correio eletrônico, ambiente virtual de aprendizagem – AVA) que tornasse viável a comunicação e garantisse a continuidade do ensino. Até então, a modalidade EAD e as tecnologias da informação e comunicação (TCI) ocupavam espaço e função marginal no sistema educacional nacional. Havia escassez de investimento no aparato tecnológico e de formação continuada para docentes utilizarem os ambientes virtuais para ensino^12^.

Em 2020, a Secretaria de Educação do Estado de São Paulo (SEDUC-SP) lançou o Centro de Mídias da Educação de São Paulo (CMSP), cujo objetivo era assegurar a manutenção das aulas, sem grande prejuízo ao calendário letivo, no período de confinamento social. O CMSP reúne um espaço de aprendizagem virtual disponibilizado no formato de aplicativo para dispositivos móveis como celulares e *tablets*, além de um sistema de transmissão por televisão em canais abertos para o estado de São Paulo. As/Os estudantes teriam a possibilidade de acessar suas respectivas aulas e atividades e interagirem ao vivo entre si e com professoras/es por meio do aplicativo. Aquelas/Aqueles que não possuíssem um dispositivo móvel poderiam assistir as aulas de todas as disciplinas diretamente pela televisão nos horários específicos. Assim, ambos os acessos dispõem de aulas síncronas e assíncronas ministradas por professoras/es da rede pública.

As/os participantes da pesquisa consideraram a experiência do ensino remoto, incluindo o Centro de Mídias, bastante frustrante e precária: foram constantes e enfáticas as críticas à desconexão entre os conteúdos e à falta de interação entre professoras/es e estudantes neste aplicativo. Majoritariamente, consideraram estudar como o maior desafio do período pandêmico (mais até do que sobreviver). Ainda, mais do que a desconexão entre professor/a-aluno/a, pesou a transferência do contexto de ensino-aprendizagem para o âmbito doméstico.

O ensino remoto expôs e intensificou desigualdades sociais associadas à exclusão ou letramento digital que afetam segmentos mais desfavorecidos economicamente, a exemplo da falta de dispositivos tecnológicos como computador, *tablet* ou *smartphones* com processador e memória adequados e o acesso precário (ou inexistente) à internet^13^. Algumas/alguns estudantes relataram não ter conseguido estudar por não possuírem acesso à internet e/ou aparelho celular – “não consegui estudar, me passaram por dó” (Vanessa, 15 anos). Essas barreiras às boas condições de estudo não foram suficientemente atenuadas pela rede de ensino, sem investimento necessário para compra de computadores, *notebooks* e instalação de banda larga compatível. Ainda que a escola tenha permanecido aberta em alguns períodos, adolescentes que a frequentaram para acompanhar as aulas online relataram dificuldades em assistir a estas por não existirem equipamentos suficientes (computadores e *notebooks*) nem rede de internet (Wi-Fi) adequada para atender às demandas no primeiro ano da pandemia (ano de 2020).

A centralização da educação online nos aplicativos flexibilizou os deveres estudantis: esfumou, por exemplo, obrigações com o cumprimento de prazos de entrega de trabalhos, fundamental para a constituição de uma disciplina no estudo e da efetivação da prática do estudar. Mostraram-se igualmente problemáticos os conteúdos genéricos, distanciados dos conhecimentos das/os estudantes e objetivamente desprovidos de contextualização, oferecidos nas aulas promovidas pela secretaria estadual de educação. O ensino padronizado trouxe-lhes dificuldade acrescida, por não estar compatibilizado com o nível de aprendizagem delas/es. As/Os estudantes também apontaram diferenças de linguagem e conteúdo em relação à habitual comunicação e temas apresentados por suas/seus professoras/es enquanto barreiras ao aprendizado. Conforme os relatos, fica patente uma confusão quanto às atividades e às várias ferramentas e plataformas de comunicação digital que foram sendo empregadas ao longo do “confinamento” pandêmico.

Outro aspecto era a impossibilidade de as/os professoras/es acompanharem o desenvolvimento das/os alunas/os, visto que não tinham acesso às atividades feitas no Centro de Mídias. A condução do ensino à distância pelo governo foi considerada “muito ruim”, já que não havia um desenvolvimento paulatino de aspectos, elementos e contextos relacionados aos temas em estudo, como feito pelas/os professoras/es da escola. Nas aulas *online*:

“vão direto ao ponto… eles querem é logo o resultado… pergunta aqui e quer que responda ali. Eles estão fazendo como se nós tivéssemos estudado antes. Não havia uma recapitulação na aula seguinte como acontece na escola. A interlocução em sala propicia ao professor perceber que a/o aluna/o não entendeu, volta o conteúdo anterior, até eles entenderem” (Téo, 15 anos).

Estar em casa em tempo integral parece ter amplificado as atribuições domésticas das/os adolescentes, dificultando o processo de escolarização, sobretudo para as meninas^14,15^. Ter que “cuidar do irmão, cuidar da casa” trouxe dificuldades de concentração nos estudos, com atrasos nas tarefas escolares e ainda mais “desânimo” com o contexto vivido. Esse alargamento das responsabilidades de cuidar dos irmãos menores e da arrumação da casa para as garotas assinala a permanência da tradicional divisão sexual do trabalho doméstico. Em geral, os rapazes possuíam menos tarefas, como “lavar a louça e varrer”; eles assumiam a limpeza apenas quando ficavam sozinhos em casa ou quando eram o filho mais velho com irmãos pequenos. Este fato é realçado pela forma como alguns garotos se referiram às tarefas, descrevendo-as como um “favor” pedido pela mãe, não uma obrigação consolidada.

### “A Gente Quer Abraçar o Amigo”: Rupturas na Sociabilidade Juvenil

A suspensão do ensino presencial trouxe para as/os adolescentes uma interrupção das relações sociais e vínculos importantes vivenciados no ambiente escolar: a escola é um território de proteção, alimentação e sociabilidade^16^. Antes da pandemia, Sara reunia-se com as/os amigas/os para ir até a pracinha do bairro “tomar um sorvete”, “e hoje não pode mais, tem essa distância assim. Se você [es]tiver assim [próximo] já falam ‘ó, o distanciamento!’, ‘tá muito perto um do outro!’. Então isso é chato. A gente quer abraçar o amigo. A gente não gosta de ficar abraçando nossa família dentro de casa não…” (Sara, 16 anos).

As redes sociais tiveram um papel fundamental para manter a socialização entre os pares^17,18^, mas o convívio presencial cria uma rede de afetos, abraços e conversas que o *online* não permite. As/os amigas/os constituem um significativo suporte entre as/os jovens, num processo constitutivo dessa fase da vida em que se intensifica o afastamento dos núcleos familiares e há maior aproximação do grupo de pares^19^. O enfrentamento do apartar foi “bem difícil”, pois se deu justamente em um momento do curso da vida em que as/os adolescentes possuem avidez quanto ao encontro de autonomia e identidade^19,20^. Embora pudessem utilizar as redes sociais – Instagram, WhatsApp, Facebook – para se comunicar virtualmente com amigas/os, o “preferir falar pessoalmente” era a tônica dos discursos, justificado pela necessidade de “contato físico” ou pela “pouca paciência” para a comunicação virtual, como expresso na fala “eu não tenho paciência pro WhatsApp” (Renata, 15 anos).

As/Os jovens passaram a conviver mais com familiares, que também tiveram sua rotina interrompida por conta das regras sanitárias impingidas, sobretudo, no primeiro ano da pandemia. Muitos pais e mães tiveram seus trabalhos alterados, seja porque passaram a trabalhar de forma remota, seja porque ficaram desempregados. O maior tempo de convívio com familiares foi identificado como gerador de conflitos, de estresse e de violências no ambiente familiar, o que tem implicações na relação de bem-estar e saúde das/os adolescentes^21,22^.

O período da adolescência é marcado por muitas mudanças e também experimentações. O contexto da crise sanitária da covid-19 gerou barreiras de socialização e construção de si para as/os adolescentes^19^. Essa ruptura, ocorrendo de modo abrupto, trouxe sentimentos de medo, incertezas, angústia, solidão – termos muito frequentes nas narrativas delas/es. “A pandemia afastou as pessoas, gerou solidão e vazio porque ficava presa, então ficava um clima mais estranho, escuro, pesado. E a gente acaba ficando mal” (Luiza, 16 anos). Em outro GF, algumas das participantes relatam a centralidade de sentimentos de incerteza e medo:

Moderadora: Como é que você acha que a pandemia colaborou nessa crise de ansiedade?

Yara (15): Não ver se vai acabar isso, não saber se um dia vai acabar, se um dia a gente vai parar de usar máscara.

Simone (16): Não saber o futuro.

Yara (15): Agora que eu tomei a vacina já tô mais de boa, mas antes eu ficava tipo: “meu Deus será que vai ter vacina? Será que o mundo vai acabar? Vai todo mundo morrer?”

Renata (15): É, “será que eu vou pegar covid? Será que… não sei o quê…?”

Yara (15): “Será que se eu pegar covid eu vou morrer?”

Renata (15): Não, não era nem comigo pegar [a] covid; tipo, minha mãe… Minha mãe tem asma, tem... tem tudo né? E aí, “e se eu passar para minha mãe? Ela vai morrer?” Porque eu pegar, beleza, mas aí minha mãe? Meu pai? Meu irmão? Minha vó?

A evocação iterativa das palavras “presa/o”, “sozinha/o” e “solidão” e da frase “não teve ninguém pra conversar” dá o tom da forma como grande parte das/os adolescentes experienciou o período de distanciamento físico-social. A condição de estar sozinha/o é uma resultante não intencional das medidas de contenção em período prolongado, vulnerabilizando o grupo e aumentando o risco de desenvolvimento de problemas de saúde mental^23^. Mesmo quando as relações familiares não são marcadas por conflitos, estas não costumam suprir a ausência dos amigos, pois amizade envolve afinidade, interesses e estilos comuns.

Autores argumentam acerca da necessidade de atenção especial aos abalos resultantes do distanciamento físico-social e da suspensão de atividades presenciais para as/os adolescentes^24,25^. A antiga rotina escolar ou com atividades fora de casa foi substituída por uma em que o “tédio” e o “desânimo” predominaram. Relatos recorrentes entre as/os adolescentes incluem: “eu só dormia e comia”, “via TV”, “mexia no celular”, “jogava”, além do aumento das tarefas domésticas. As mudanças percebidas também dizem respeito à menor produtividade, aumento do sedentarismo, adoção de hábitos alimentares menos favoráveis à saúde e rotina de sono desregulada^23,26^. Pesquisa realizada junto a escolares do nono ano do nível fundamental, matriculadas/os em unidades de ensino da Região Metropolitana de São Paulo, registrou o tempo de exposição às telas, a inversão do sono e ser do sexo feminino como elementos associados a sintomas de depressão e ansiedade entre as/os participantes do estudo^26^.

Nesse ponto, importa localizar a centralidade da escola como espaço de sociabilidade e de construção de vínculos afetivos e emocionais entre amigos. Questões de sofrimento psicossocial^7^ relacionadas aos problemas familiares (relações conflituosas/violentas, negligentes e distanciamento afetivo) estão presentes nas narrativas. O afastamento da escola significa o afastamento de amigas/os, do contato, da presença, do apoio de colegas. Companheiras/os de turma consubstanciam uma rede de solidariedade que fornece contínuo apoio emocional e afetivo. A “turma” é capaz de perceber dias de tristeza e desesperança e tenta alegrar o/a companheiro/a com conversas e brincadeiras. Esse laço se fortalece à medida que vão se conhecendo no avançar dos anos escolares juntos. Os momentos de interajuda operam como espaço de promoção de cuidado e liberdade de posicionamento; têm relevância educativa ao estimular habilidades sociais e a construção de sentidos acerca de suas crenças, aspirações, medos, ansiedades – sentimentos estreitamente relacionados à construção da identidade^27^.

Guardar para si as adversidades pode exacerbar a desilusão, ideações suicidas, sintomas de depressão e a própria sensação de solidão – dimensões presentes nos discursos das/os participantes dos GF e em outros momentos de interação, inclusive pré-pandemia, feitos entre pesquisadoras do estudo e alunas/os desta mesma escola^28^. As frequentes narrativas sobre sofrimento psicossocial parecem reiterar o argumento de Najar e Castro^29^ que, recuperando um outro autor, Frank Furedi, falam sobre a psicologização da vida cotidiana e um maior senso de insegurança existencial como alguns dos elementos de um certo “script cultural” que estaria influenciando as reações à covid-19. Para estes autores, “a maneira como um grupo social responde a uma ameaça, tal como uma pandemia, é mediada pela percepção da ameaça, seu senso de segurança existencial e a capacidade de dar sentido às experiências imprevisíveis” (p. 145).

### O “Maior Experimento Psicológico do Mundo”: Sentimentos Diante do Extraordinário

O estado de contingência social instaurado pela pandemia agravou as condições do sentimento de ansiedade preexistentes. As incertezas, o “não saber o futuro”, rompem com perspectivas de projeto de vida. O “medo”, somado à “preocupação” excessiva, e o ficar “sozinho/a” são constantes vivenciadas e relatadas pelas/os adolescentes. As jovens ofereceram relatos mais pormenorizados acerca desses sentimentos, enquanto os garotos restringiram-se a expressar “raiva” e “ódio” pela desestruturação de sua rotina escolar e interação com os amigos. Relatos e situações semelhantes são encontrados em outros estudos com adolescentes e jovens: o estar “confinado” imposto pela pandemia foi descrito pelos/as participantes deste estudo, bem como no estudo de Lima^30^, como o “maior experimento psicológico do mundo”, colocando em xeque a capacidade humana de encontrar algum sentido em meio a tanto sofrimento e desafios, individuais e coletivos.

Inseridas em um espaço domiciliar e com ausência de atividades de lazer, algumas das adolescentes relataram que dormiam para passar o tempo e evitar o tédio. Afirmaram ainda que ficaram “mais nervosas” durante a pandemia e que engordaram – “Ganhei uns 10 kg na quarentena” (Vanessa, 15 anos); “Eu ganhei 11 kg” (Mônica, 15 anos). A carência de opções de distração dentro de casa, aliada ao decréscimo de atividades físicas, ao aumento do sedentarismo, ao “estresse” e à “ansiedade”, ajuda a compreender o aumento de peso referido pelas meninas^24^.

Alguns rapazes possuem relatos semelhantes aos das garotas, como dormir ou ficar no quarto jogando o dia todo. No entanto, há aqueles que explicitaram repetidas quebras do período de distanciamento físico para estar com amigos e colegas próximos (vizinhos) ou namoradas/ficantes, ressaltando que o fizeram quando houve redução no número de casos de infecção e de mortes. Cabe pontuar que as medidas não farmacológicas, muitas vezes, tiveram adesão por parte das/os jovens pela exigência e vigilância das mães. Todavia, aquelas/es cujas mães permaneceram em trabalho presencial não cumpriram à risca o período de distanciamento: a ânsia pelo convívio fez com que houvesse escape do isolamento.

Ainda, a narrativa juvenil associa o medo constante e a insegurança sobre o adoecimento e morte dos familiares a sentimentos de depressão e ansiedade. Esse tipo de associação é apontado pela literatura específica sobre saúde mental: estudos indicam que adolescentes que tiveram pais em trabalhos considerados essenciais, ou que não puderam fazer o distanciamento físico, tiveram mais sintomas de sofrimento psíquico. Similarmente, adolescentes que viviam em família com condições econômicas vulneráveis apresentaram maiores taxas de estresse^23,31^.

A convergência dos efeitos deletérios decorrentes da pandemia em curso tem repercussões negativas em diversas esferas da vida, tendo relevo os aspectos associados ao sofrimento psicossocial^7,30^. As medidas de distanciamento físico trouxeram preocupações acrescidas às/aos adolescentes, cuja fase da vida é marcada pela centralidade do grupo e potencialização do sentimento gregário entre pares^14^. Esse tem sido considerado importante estopim para o surgimento de problemas relacionados ao sofrimento psicossocial, potencializado pelas situações de vulnerabilidade individual, social e programática das/os adolescentes frente ao caótico cenário pandêmico^7^.

## CONSIDERAÇÕES FINAIS

As medidas de distanciamento físico-social e o fechamento das escolas trouxeram prejuízos ao processo de escolarização e socialização das/os jovens, bem como limitaram importantes oportunidades do convívio entre pares, com impacto tanto no desenvolvimento de identidade, autonomia e independência das/os adolescentes quanto na manutenção da saúde física e psicossocial. Este estudo documenta como medidas de controle da pandemia, implementadas de forma fragmentada e sem suporte às famílias mais empobrecidas, têm efeitos negativos em outras esferas da vida, em particular para jovens pobres.

Esta pesquisa mostra as implicações e fragilidades das/os adolescentes moradores de uma região periférica e muito vulnerável em uma grande metrópole latino-americana. O contexto social mostrou-se determinante para a experiência desse grupo de viver a pandemia de covid-19, desde a adesão intermitente às medidas não farmacológicas de prevenção até a própria impossibilidade de executar o distanciamento social, uma vez que muitos tinham familiares que estavam trabalhando em serviços considerados essenciais, com necessidade do uso de transportes públicos lotados na fase mais aguda da pandemia.

As/os adolescentes relataram sentimentos de depressão e ansiedade, gerados pela angústia e medo provocados pela pandemia da covid-19. Muitos falaram da relação com as/os amigas/os e da rede de apoio que elas/es representam. Efeitos diretos na saúde, como sedentarismo e aumento de peso, foram mencionados. Entretanto, ainda é cedo para compreender integralmente os efeitos da pandemia e as implicações a longo prazo das estratégias de controle e prevenção da doença adotadas no Brasil, país que figura dentre os que tiveram mais casos e óbitos por covid-19 no mundo^32^.

A ONU assinala que os países devem assegurar o acesso seguro à educação de alta qualidade nas escolas durante emergências com a mesma atenção destinada aos serviços de saúde^33^. A escola se mostra como território de sociabilidade, de apoio e de refúgio para lidar com os problemas de ordem familiar e pessoais, além de cenário de identificação de abusos e negligências. Ao que parece, é também ali um lugar privilegiado para se propor/construir ações que auxiliem estas/es adolescentes a lidar com as feridas deixadas pela pandemia. “Nas áreas periféricas, tanto vulnerabilidades quanto oportunidades têm, em grande medida, bases territoriais”^26^. Ainda que acreditemos na resiliência e plasticidade de crianças e jovens, esta pesquisa indica que o caminho para a recuperação do ensino e da sociabilidade juvenis será longo e merece atenção redobrada^34^. Este cenário demanda esforços, sobretudo em termos de proposições de políticas públicas para o enfrentamento das diversas desigualdades sociais (incluindo as de gênero, classe social, raça/etnia, regiões de moradia) acirradas pela pandemia, bem como em relação às possibilidades de recuperação (*recovery*)^33^, considerando a dinâmica local da pandemia.
